# A Novel Role of E-Cadherin-Based Adherens Junctions in Neoplastic Cell Dissemination

**DOI:** 10.1371/journal.pone.0133578

**Published:** 2015-07-24

**Authors:** Svetlana N. Rubtsova, Irina Y. Zhitnyak, Natalya A. Gloushankova

**Affiliations:** Institute of Carcinogenesis, N.N. Blokhin Russian Cancer Research Center, Moscow, Russia; Wayne State University School of Medicine, UNITED STATES

## Abstract

Using confocal microscopy, we analyzed the behavior of IAR-6-1, IAR1170, and IAR1162 transformed epithelial cells seeded onto the confluent monolayer of normal IAR-2 epithelial cells. Live-cell imaging of neoplastic cells stably expressing EGFP and of normal epithelial cells stably expressing mKate2 showed that transformed cells retaining expression of E-cadherin were able to migrate over the IAR-2 epithelial monolayer and invade the monolayer. Transformed IAR cells invaded the IAR-2 monolayer at the boundaries between normal cells. Studying interactions of IAR-6-1 transformed cells stably expressing GFP-E-cadherin with the IAR-2 epithelial monolayer, we found that IAR-6-1 cells established E-cadherin-based adhesions with normal epithelial cells: dot-like dynamic E-cadherin-based adhesions in protrusions and large adherens junctions at the cell sides and rear. A comparative study of a panel of transformed IAR cells that differ by their ability to form E-cadherin-based AJs, either through loss of E-cadherin expression or through expression of a dominant negative E-cadherin mutant, demonstrated that E-cadherin-based AJs are key mediators of the interactions between neoplastic and normal epithelial cells. IAR-6-1DNE cells expressing a dominant-negative mutant form of E-cadherin with the mutation in the first extracellular domain practically lost the ability to adhere to IAR-2 cells and invade the IAR-2 epithelial monolayer. The ability of cancer cells to form E-cadherin-based AJs with the surrounding normal epithelial cells may play an important role in driving cancer cell dissemination in the body.

## Introduction

Classical cadherins are transmembrane glycoproteins that mediate cell-cell adhesion through Ca^+2^-dependent homophilic trans-interactions of their ectodomains, forming adherens junctions (AJs) [1]. In AJs, intracellular domains of cadherins interact with catenins, which in turn interact with actin and several actin-binding proteins. Actin filaments are essential for the stability of AJs. E-cadherin plays a key role in the maintenance of stable cell-cell adhesion in epithelial tissues. For many years, epithelial-mesenchymal transition (EMT) has been regarded as the trigger for invasion and metastasis of carcinoma cells [2]. Down-regulation of E-cadherin and weakening of cell-cell adhesion are considered crucial steps in EMT [3–5]. Immunohistochemical studies showed that carcinomas often lose E-cadherin expression. It has been generally accepted that in human malignancies, reduced expression of E-cadherin was associated with infiltrative growth, metastasis and poor prognosis for the patient [6, 7]. Many invasive carcinomas (ductal breast carcinomas, inflammatory breast carcinomas, colorectal carcinomas, prostate carcinomas, and oral squamous cell carcinomas), however, retain expression of E-cadherin and its accumulation at the plasma membrane [8–12]. This suggests that invasion of carcinoma cells into the adjacent tissues is not prevented by the presence of E-cadherin. A critical analysis of studies evaluating E-cadherin immunoexpression in carcinomas puts into question the association of reduced immunohistochemical staining of E-cadherin with poor prognosis. There is considerable heterogeneity in the intensity of immunohistochemical staining of E-cadherin between different tumors and within the same tumor. The differences in immunohistochemical techniques used to detect alterations in E-cadherin expression, in the choice of the E-cadherin specific antibody, and in the methods of assessment of E-cadherin-positive or E-cadherin negative cases may also contribute to the discordant interpretations of the results of immunostaining [12, 13].

The role of E-cadherin in cancer biology may be more complex and tumor type specific. Not only a suppressive but also a positive role for E-cadherin in neoplastic progression has been discussed. E-cadherin and E-cadherin-based AJs may be important for tumor cell survival, growth, invasion and metastasis [14–16]. In ovarian carcinoma, ectopic E-cadherin expression may have a survival effect on cancer cells joined by the AJs into tumor aggregates when they float in the peritoneal cavity [17]. It was also found that in distant metastases of some carcinomas E-cadherin expression was stronger than in the primary tumor. Re-expression of E-cadherin in metastases from primary tumors where E-cadherin was down-regulated was also described [18, 19].

Many invasive carcinomas may infiltrate surrounding tissues as multicellular clusters in which tumor cells retain E-cadherin-based AJs. This process is known as collective migration [20] and is prevalent in carcinomas from breast, prostate, and colon, and in squamous cell carcinomas [14, 21, 22]. We have previously shown that neoplastic transformation by mutated Ras or chemical carcinogenes results in reorganization of E-cadherin-based AJs in cultured IAR epithelial cells. E-cadherin-based radial AJs in transformed cells were very dynamic und unstable [23]. These AJs had important roles in collective migration of transformed IAR epithelial cells over 2D substrates and in migration chambers [24].

In the present study, we analyzed the behavior of transformed epithelial cells seeded onto the confluent monolayer of normal epithelial cells and discovered a novel role for E-cadherin-based AJs in neoplastic cell dissemination. Our findings show that neoplastic cells retaining the expression of E-cadherin are able to establish E-cadherin-based adhesions with normal epithelial cells. These AJs provide the adhesion of transformed cells to normal cells, and are necessary for their migration over the epithelial monolayer and invasion of the monolayer.

## Materials and Methods

### Cell lines, constructs and transfections

The IAR-2 line of non-transformed rat epithelial cells and the IAR-6-1 line of IAR epithelial cells transformed with dimethylnitrosamine in vitro were established by Montesano et al. at the International Agency for Research on Cancer [25]. Using retroviral infection, the IAR-2 line was transformed with N-RasG12D [23]. Individual clones of Ras-expressing cells—IAR1162-D3, IAR1162-F4, IAR1162-C4, IAR1170-D11, IAR1170-F9, and IAR1170-H5—were established [23]. The IAR-6-1 line expressed E-cadherin but not N-cadherin, all IAR1170 clones expressed both E- and N-cadherin, IAR1162-D3 and IAR1162-F4 clones expressed N-cadherin but not E-cadherin, and IAR1162-C4 clone did not express either E- or N-cadherin. The tumorigenicity of established clones has been previously described [24]. The EGFP and mKate2 constructs were purchased from Evrogen (Russia). Plasmids expressing GFP-E-cadherin, E-cadherin cDNA, and the dominant negative E-cadherin mutant were kindly provided by S. Troyanovsky (Northwestern University, Chicago, IL, USA). The dominant-negative mutant form of E-cadherin carried a W156A mutation in the Ec1 domain that abolished adhesive E-cadherin interactions [26]. IAR-6-1DNE-E10 and IAR-6-1DNE-H9 clones that expressed the dominant negative E-cadherin mutant were established. IAR1162-D3 clone was transfected with the plasmid containing E-cadherin cDNA, and the IAR1162-D3E line expressing exogenous E-cadherin was established. Transfections were performed with the LTX Lipofectamine transfection reagent (Life Technologies) according to the manufacturer’s protocol. Clones were obtained after 2 weeks of selection with G418 (1μg/ml).

IAR-6-1, IAR-6-1DNE-E10, IAR-6-1DNE-H9, IAR1162-D3, and IAR1162-D3E were infected with a lentivirus containing GFP cDNA. Briefly, 293T packaging cells were co-transfected with three plasmids: pCMV-dR8.2 dvpr, pCMV-VSV-G and pGIPD P2 using the Lipofectamine 2000 transfection reagent (Life Technologies) according to the manufacturer’s protocol. 48 hours post transfection, conditioned medium containing virus stock was collected, passed through a 0.22 μm pore filter, and added to IAR cells for 6 hours. Clones were obtained after 5 days of selection with puromycin (1 μg/ml). pCMV-dR8.2 dvpr (Addgene plasmid # 8455) and pCMV-VSV-G (Addgene plasmid # 8454) were a gift from R.A. Weinberg (Whitehead Institute for Biomedical Research, Cambridge, MA, USA) [27]. pGIPD P2 plasmid, which is a modification of lentiviral pGIPZ vector containing TurboGFP and PuroR (Open Biosystems), was a gift from D. Mazurov (Institute of Immunology, Moscow, Russia) [28].

### Double layered cell culture

Normal IAR-2 epithelial cells expressing mKate2 or GFP-E-cadherin were seeded at high density (0.7–1.0x10^6^ cells per dish) to form a confluent monolayer 24 hours after seeding. At this time point, transformed cells expressing GFP, mKate, GFP-E-cadherin, the dominant negative E-cadherin mutant in combination with GFP, or GFP expressing transformed cells which had been transfected with various siRNAs 48 hours earlier were seeded at low density (0.5–1.25x10^5^ cells per dish) onto the IAR-2 monolayer. Data were collected at 16–24 hours after seeding of the transformed cells.

### Live-cell imaging

For live-cell imaging, cells were seeded into 35-mm glass bottom culture dishes (MatTeck Corporation). 20 min before imaging, cells were transferred into phenol red-free DMEM/F-12 medium with L-glutamine and HEPES (Sigma) supplemented with 10% FCS (PAA Laboratories). Cells were observed with a Leica TCS SP5 confocal laser scanning microscope (HDX PL APO 40x 1.25 or HDX PL APO 63x 1.4) or with a Nikon Eclipse Ti-E microscope (Plan 20x B/CN; ORCA-ER camera by Hamamatsu Photonics; NIS-Elements AR 2.3 software by Nikon). For “Z-projections”, three Z-slices were overlaid using Adobe Photoshop CS2 software with two upper slices as layers in “screen” mode. For tracking, the position of the cell’s center of mass throughout a time lapse sequence was determined and the results were plotted onto one of the frames of the sequence using Fiji/ImageJ with the Manual Tracking plugin (Fabrice P. Cordelières, Institut Curie, Orsay, France).

### Transepithelial migration assay

The double layered cell cultures described above (various EGFP-expressing transformed IAR cells + mKate-expressing normal IAR-2 cells) were established in glass bottom culture dishes. Two confocal slices (at the glass substrate level beneath the monolayer and at 10 ± 2 μm above the substrate, over the monolayer) were obtained. The number of transformed cells that had invaded the monolayer and spread on the glass substrate was counted in 30 fields. All measurements were reported as mean ± standard error of the mean. All studies were performed in triplicate.

### Antibodies and Reagents

The following antibodies were used: mouse monoclonal anti E-cadherin, clone 36 (BD Transduction Labs), final dilution 1:100; mouse monoclonal anti-actin beta, clone 4C2 (AbD Serotec Bio-Rad), final dilution 1:100; TRITC-conjugated goat polyclonal anti-mouse IgG, code 115-025-166 (Jackson Immunoresearch), final dilution 1:100; Alexa Fluor 647-conjugated goat polyclonal anti-mouse IgG2a, code 115-605-206 (Jackson Immunoresearch), final dilution 1:200; Alexa Fluor 488-conjugated goat polyclonal anti-mouse IgG1, code 115-545-205 (Jackson Immunoresearch), final dilution 1:200, and horseradish peroxidase-conjugated goat polyclonal anti-mouse IgG antibodies (Chemicon), final dilution 1:3000. Other reagents were obtained from Sigma-Aldrich.

### Western blot analysis

Cells were lysed with Mg2+ Lysis/Wash Buffer (Upstate EMD Millipore) including protease inhibitor cocktail (Sigma-Aldrich), 0.25mM Na_3_VO_4_, 1mM DTT, 10mM NaF. Samples were heated for 10 min at 95°C and loaded onto 10% SDS-polyacrylamide gel in equal protein concentrations according to the SDS-PAGE Bio-Rad protocol. Resolved proteins were transferred to Amersham Hybond-P PVDF membranes (GE Healthcare). Membranes were blocked with 5% m/v solution skim milk powder (Fluka Sigma-Aldrich) in PBS with 0.1% v/v of Tween 20 (AppliChem) for 1 h followed by incubation with the primary antibodies at 4°C overnight. After washing, peroxidase-conjugated secondary antibodies were applied for 1 h at 37°C. Blotted protein bands were detected using Pierce ECL Western Blotting Substrate (Thermo Fisher Scientific), and chemiluminescence images were captured by an image acquisition Chemi-Smart 2000 system (Vilber Lourmat) and ChemiCapt software.

### siRNA Transfection

siRNA for E-cadherin (ON-TARGETplus SMARTpool Rat Cdh1 siRNA, L-088449-02-10), N-cadherin (ON-TARGETplus Rat Cdh2 siRNA, L-091851-02-0005) and ON-TARGETplus Non-targeting siRNA (D-001810-10-05) were purchased from Dharmacon (GE Healthcare). Subconfluent cells were transfected with 20 nM of siRNA using DharmaFECT 1 Transfection Reagent according to the manufacturer’s protocol (Dharmacon GE Healthcare). The effectiveness of siRNAs was verified by Western blotting to corresponding proteins at 72 hr post-transfection.

### Fluorescent staining and microscopy

For double staining for E-cadherin and actin, cells were fixed with 1% PFA for 10 min and methanol for 2 min. For E-cadherin staining, cells were fixed with methanol/acetone 1:1 at -20°C for 10 min. Fixed cells were incubated for 40 min with primary antibodies and subsequently for 40 min with fluorochrome-conjugated secondary antibodies. Mounted samples were examined with a Leica TCS SP5 confocal laser scanning microscope equipped with an HDX PL APO 100x 1.4 objective or a Nikon Eclipse Ti-E microscope equipped with a Plan Fluor 40x objective and a cooled CCD ORCA-ER camera (Hamamatsu Photonics) controlled via Nis-Elements AR 2.3 software (Nikon).

## Results

### Behavior of transformed epithelial cells on the confluent monolayer of normal epithelial cells

**Fig 1:**
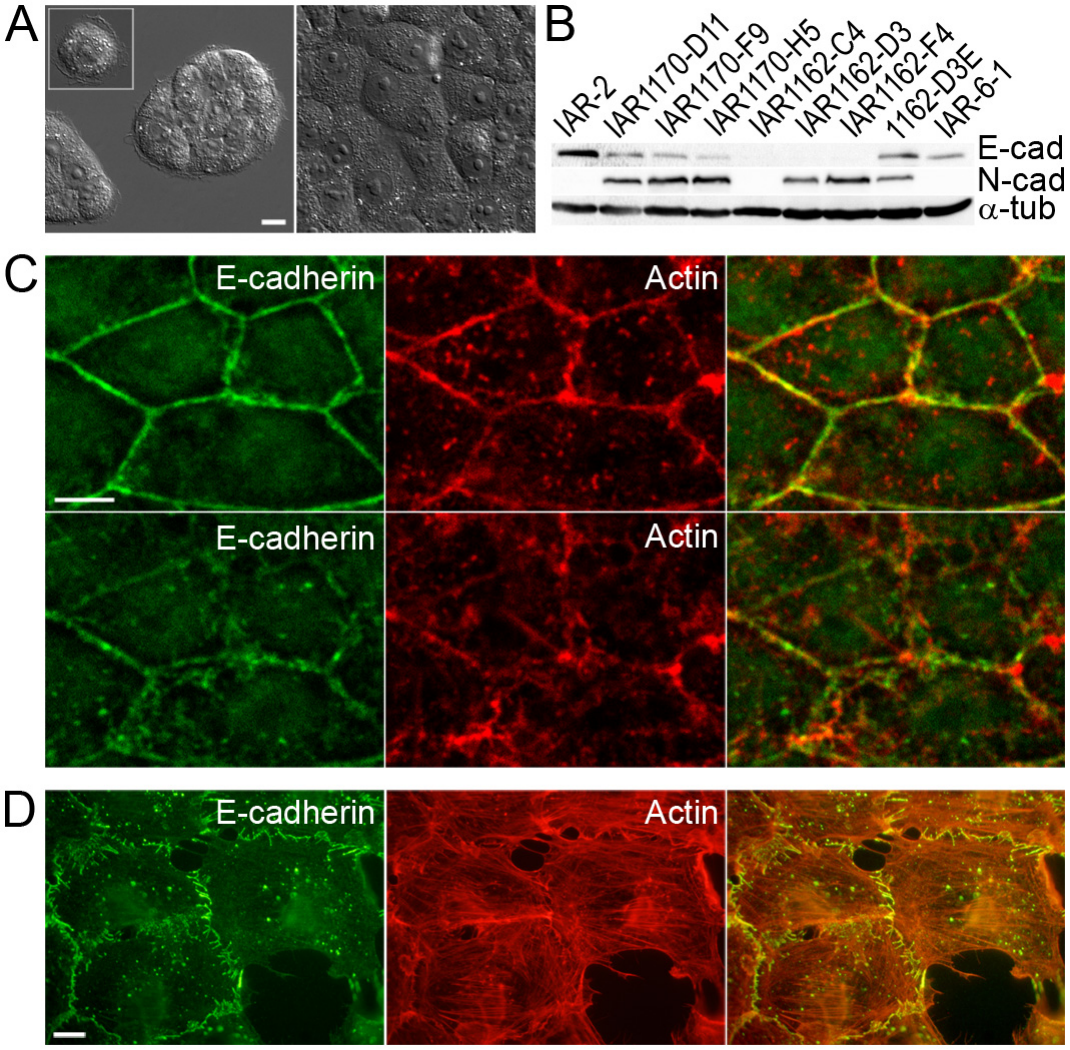
Normal and transformed IAR epithelial cells. (A) Single IAR-2 epithelial cells are discoid, they form islands in sparse culture and a confluent monolayer in dense culture. Scale bar 10 μm. (B) E-cadherin and N-cadherin expression in normal and transformed IAR cells. (C) IAR-2 cells were stained for E-cadherin (green) and actin (red). Top row (1.25 μm above the substrate) shows AJs organized as adhesion belts encircling each cell and co-localizing with circumferential actin bundles in the apical parts of cells. Bottom row (substrate level) shows parts of adhesion belts and irregular distributions of actin. Scale bar 10 μm. (D) Transformed IAR-6-1 cells were stained for E-cadherin (green) and actin (red). In these cells, radial AJs were associated with thin actin bundles. Scale bar 10 μm.

Here we investigated the motile behavior of neoplastic epithelial cells seeded onto the confluent monolayer of normal epithelial cells. We used immortalized cells of the IAR-2 line established from rat liver at the International Agency for Research on Cancer [25]. Individual IAR-2 cells were discoid-shaped, in sparse culture, they formed islands and in dense culture, a confluent monolayer (Fig 1A). IAR-2 cells expressed E-cadherin and did not express mesenchymal N-cadherin (Fig 1B). The E-cadherin-based AJs in IAR-2 cells aligned as continuous belts along the cell-cell boundaries, and co-localized with the circumferential actin bundles (Fig 1C). These AJs were very stable and dissolved only during mitosis. IAR-2 cells did not induce tumor growth upon introduction into nude mice (S1 Fig) or syngeneic rats [25]. In all aspects, immortalized IAR-2 cells looked and behaved like normal epithelial cells, and will be considered as such in the present study (S1 Video and S1 Table). We also used transformed descendants of IAR cells—IAR-6-1 line transformed with dimethylnitrosamine, and IAR1170-D11, IAR1170-F9, IAR1170-H5, IAR1162-C4, IAR1162-D3, and IAR1162-F4 clones transformed with N-RasG12D. Expression of E- and N- cadherin in these clones is shown at Fig 1B.

**Fig 2:**
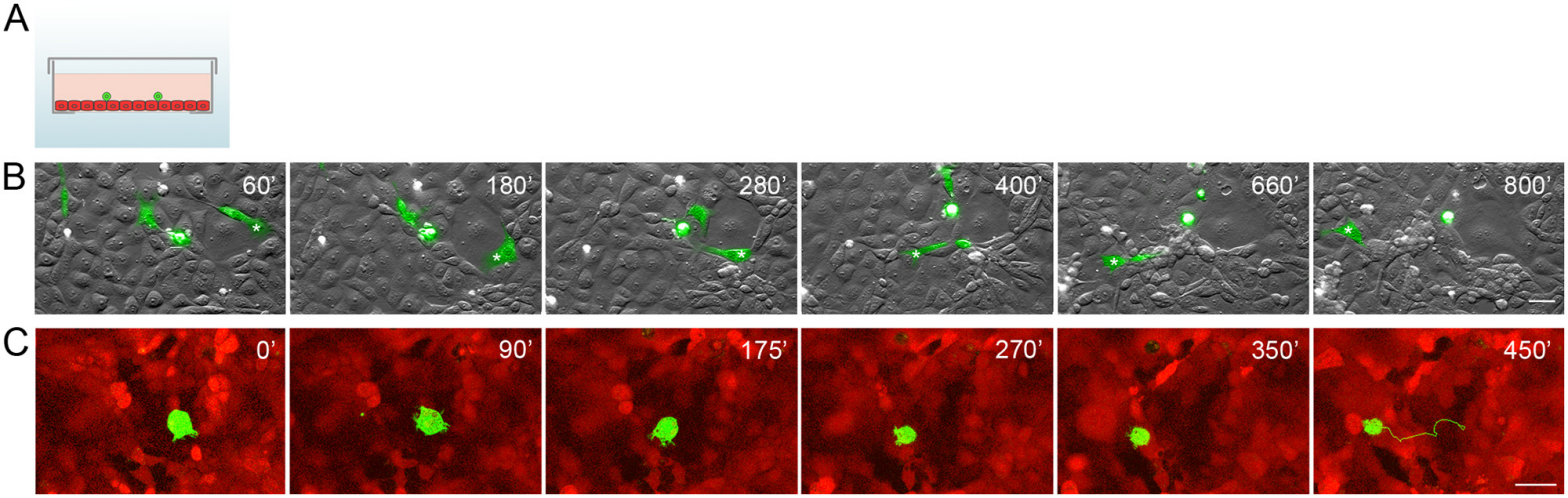
Transformed IAR-6-1 epithelial cells migrate over the monolayer of normal IAR-2 epithelial cells. EGFP-expressing IAR-6-1 cells were seeded onto the confluent monolayer of mKate2-expressing IAR-2 cells. (A) A scheme of experimental design used in the present study: a glass bottom culture dish with a confluent IAR-2 monolayer (red) and transformed IAR cells (green) seeded sparsely onto the monolayer. (B) Selected frames from S2 Video with combined DIC and green channels. Asterisks indicate migration of an elongated fibroblast-like cell. Scale bar 40 μm. (C) Selected frames from S3 Video with combined red and green channels of the top confocal slices out of time lapse Z-stacks. A corresponding 450-min track (1 point/15 min) of the migrating IAR-6-1 cell is shown on Frame 6. Scale bar 20 μm.

Using time-lapse videomicroscopy, we investigated the behavior of transformed epithelial cells seeded onto the confluent monolayer of IAR-2 normal epithelial cells (Fig 2A). To distinguish between neoplastic and normal cells, we established stable lines of IAR-6-1, IAR1162, and IAR1170 cells expressing EGFP and IAR-2 cells expressing mKate2.

First we investigated the interactions between IAR-6-1 cells that expressed E-cadherin but did not express N-cadherin, and IAR-2 cells. IAR-6-1 cells that were seeded onto the IAR-2 monolayer, adhered to normal IAR-2 cells. IAR-6-1 cells on the monolayer were mostly round or polygonal with several protrusions that extended out from the cell body, in some cases the cells had an elongated fibroblast-like phenotype. By extending protrusions and retracting the rear, IAR-6-1 cells were able to migrate over epithelial monolayer (Fig 2B and 2C, S2 and S3 Videos). The migration pattern was uneven, with bursts of migration interspersed with periods of relative inactivity.

### Transformed epithelial cells form E-cadherin-based AJs with normal epithelial cells

**Fig 3:**
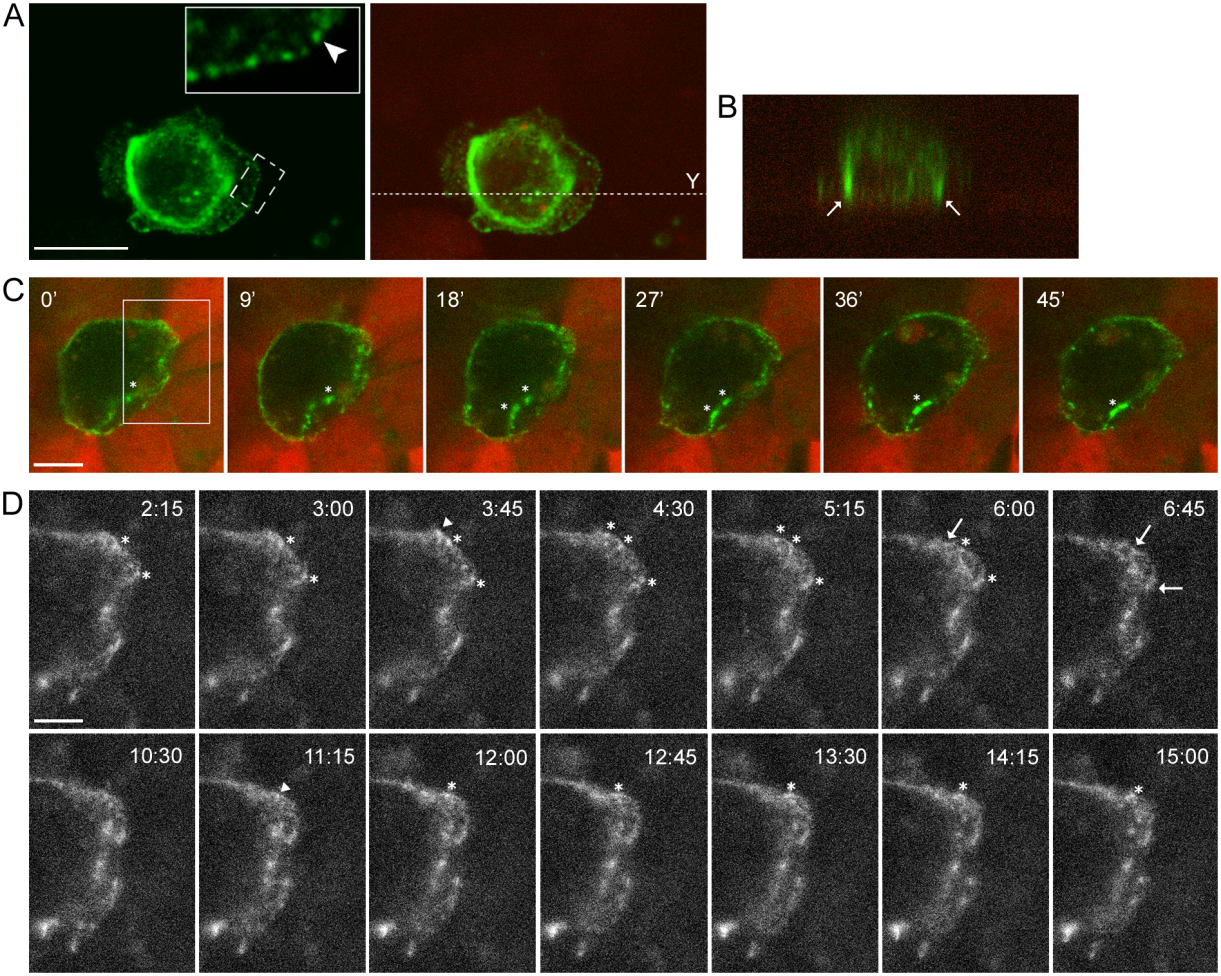
Transformed IAR-6-1 cells form E-cadherin-based AJs with underlying normal IAR-2 cells. GFP-E-cadherin-expressing IAR-6-1 cells were seeded onto the confluent monolayer of mKate2-expressing IAR-2 cells. (A-B) Immunofluorescent staining for GFP. (A) E-cadherin accumulates in dot-like adhesions at the leading edge and in prominent AJs encircling the IAR-6-1 cell. Left—green channel. Boxed region is enlarged. Arrowhead indicates dot-like adhesions. Right—green and red channels. Dotted line indicates the position of the Y-projection. Scale bar 10 μm. (B) Y-projection. Arrows mark lateral AJs between IAR-6-1 and IAR-2 cells. (C) Selected confocal slices from time lapse Z-stacks (S4 Video). The green channel is a “Z- projection” of all three slices in a confocal Z-stack, the red channel is the top slice. Asterisks indicate lateral AJs. Scale bar 10 μm. (D) A close-up view of the boxed region from (A). “Z-projection” of the green channel of the same video. At the leading edge of the IAR-6-1 cell, transient E-cadherin-based AJs are formed and quickly disassembled. Arrowheads mark spots where diffuse E-cadherin accumulates into dot-like adhesions, asterisks mark persisting E-cadherin dots, and arrows indicate disappearance of the dots. Scale bar 5 μm.

The presence of dynamic E-cadherin-based adhesions between neoplastic and normal epithelial cells was detected by live-cell imaging. For these experiments we used IAR-2 cells stably expressing mKate2. IAR-6-1 cells were stably transfected with the GFP-E-cadherin construct. GFP-E-cadherin did not affect the distribution of endogenous E-cadherin and colocalized with it in AJs (S2 Fig). In IAR-6-1 cells seeded onto the IAR-2 monolayer, E-cadherin accumulated in dot-like adhesions at the leading edge and in large AJs at the rear and at the sides of transformed cells (Fig 3A and 3B). Using live-cell confocal imaging, we found that dot-like E-cadherin-based adhesions constantly formed and disappeared at the leading edge of transformed cells (Fig 3C and 3D, and S4 Video). Larger, more stable, AJs at the sides could merge (Fig 3A and S4 Video). We hypothesize that transformed cells are capable of migrating over the normal epithelial monolayer by attaching to underlying cells with E-cadherin-based AJs and using these AJs as anchor points.

### Transepithelial migration of transformed epithelial cells

**Fig 4:**
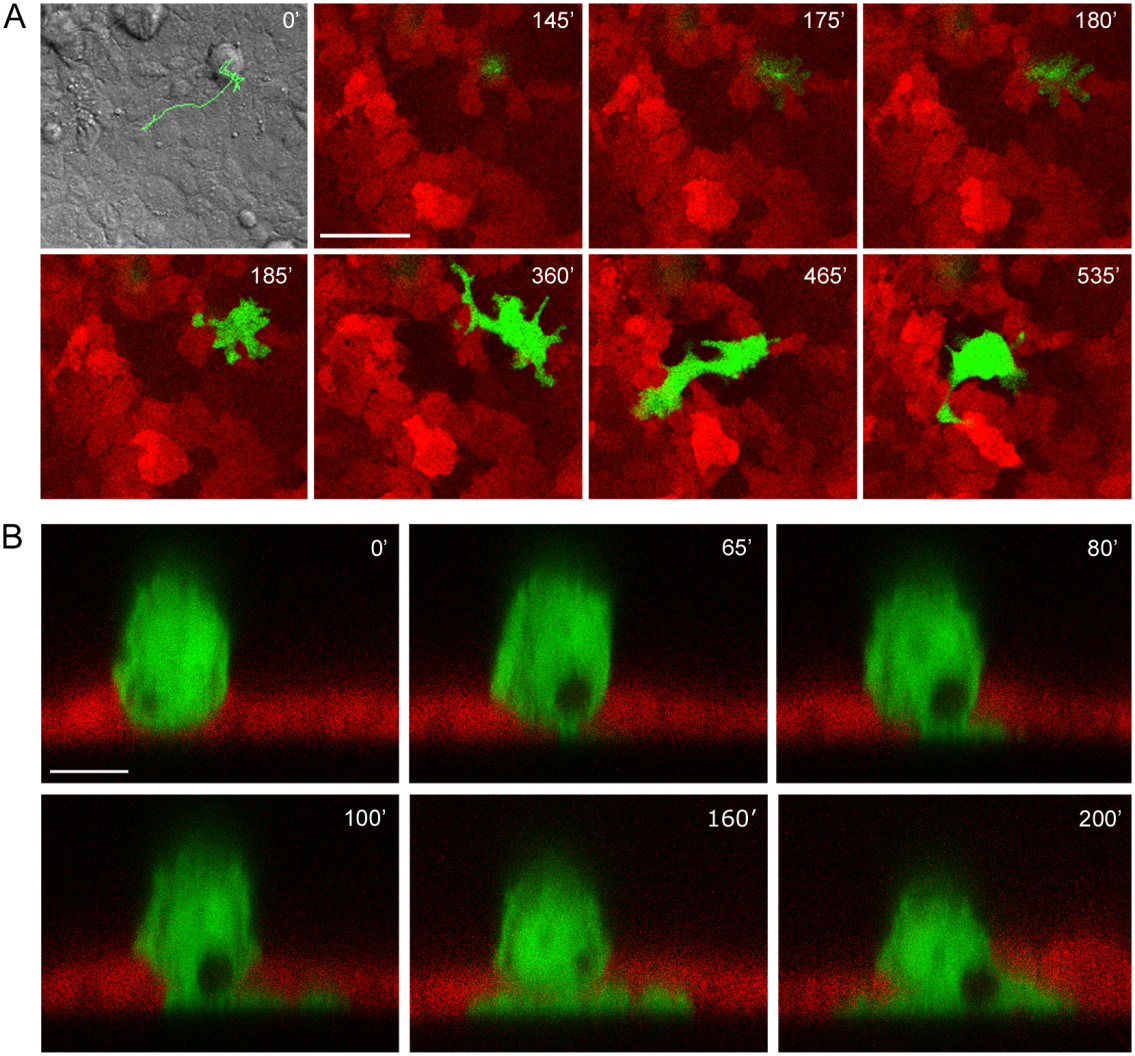
Transformed IAR-6-1 cells invade the monolayer of normal IAR-2 cells. EGFP-expressing IAR-6-1 cells were seeded onto the monolayer of mKate2-expressing IAR-2 cells. (A) Frames from S5 Video, bottom slices out of time lapse confocal Z-stacks (substrate level). Frame 1 is a DIC image of the corresponding field taken at t = 0’, with the overlaid track (525 min; 1 point/15 min) of the migrating IAR-6-1 cell. The IAR-6-1 cell is on top of the monolayer at 145’; a narrow pseudopod invades the monolayer and can be seen at the substrate level at 175’ and spreads at 180'; the entire cell migrates across the monolayer and spreads on the underlying substrate at 185’, and the cell acquires an elongated shape and migrates underneath the monolayer at 365–535’. Scale bar 50 μm. (B) Frames from S6 Video, middle slices out of time lapse confocal Y-stacks. At 0’, the entire IAR-6-1 cell is on top of the IAR-2 monolayer, cupped in the indentation in the underlying IAR-2 cell. At 65’, a narrow pseudopod extends, invading the monolayer and touching the underlying substrate. At 80–100’, the pseudopod widens and spreads across the substrate, and at 160–200’, the cell body migrates across the monolayer. Scale bar 10 μm.

We also found that by 4–8 h after seeding onto the IAR-2 monolayer, transformed cells began to invade normal epithelial cells. By 24 h after seeding approximately 15–20% of transformed IAR-6-1 cells invaded the monolayer from the top down and attached to the surface of the glass underneath IAR-2 cells. Neoplastic cells could migrate over the glass substrate underneath the epithelial monolayer (Fig 4A and S5 Video). In some cases, attachment of an IAR-6-1 cell to glass was followed by its detachment and apical extrusion from the monolayer (data not shown). Similar apical extrusion of MDCK epithelial cells with tetracycline-induced expression of RasV12 in mixed culture with normal epithelial cells was demonstrated by Hogan et al. [29].

To investigate in detail the sequence of events that occur during invasion of the epithelial monolayer by neoplastic cells, time lapse Y-stacks were acquired. The transformed cell on top of the monolayer initially formed a pseudopod that penetrated the monolayer and attached to the glass underneath IAR-2 cells. Within 1–2 hours, the whole cell body squeezed through the monolayer and spread on the glass surface (Fig 4B and S6 Video).

### Disruption of cell-cell contacts between normal epithelial cells during invasion of the monolayer by transformed cells

**Fig 5:**
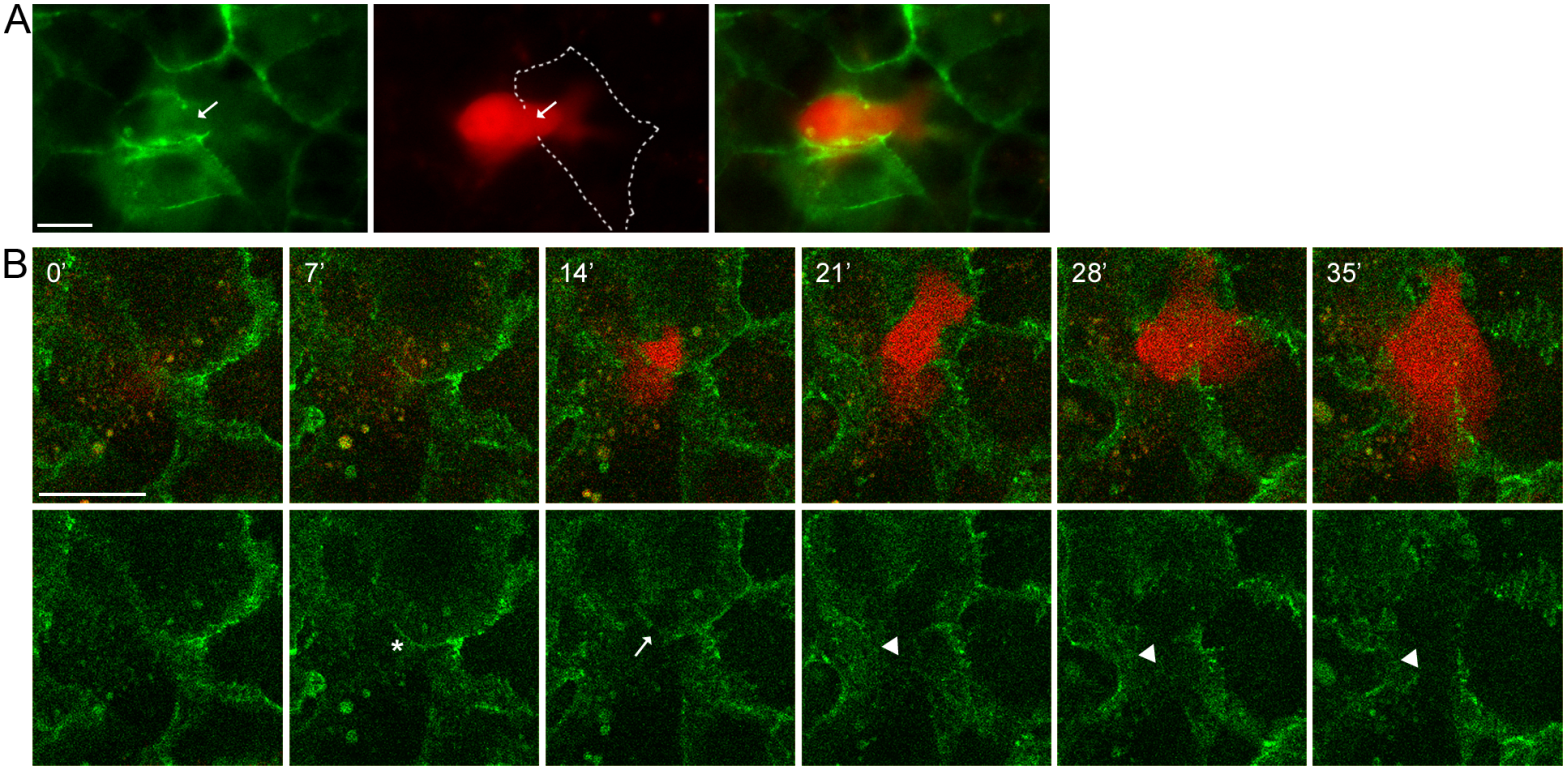
Transformed IAR-6-1 cells invade the epithelial monolayer by disrupting AJs between normal IAR-2 cells. mKate2-expressing IAR-6-1 cells were seeded onto the monolayer of GFP-E-cadherin-expressing IAR-2 cells. (A-B) Live-cell imaging. (A) An IAR-6-1 cell invades the IAR-2 monolayer at the boundary between normal cells and disrupts the AJs (arrow). Dotted line marks the AJs of an IAR-2 cell. Scale bar 10 μm. (B) Top row shows selected frames from S7 Video with combined green and red channels (bottom slices out of confocal Z-stacks, substrate level); bottom row shows the corresponding green channel images. The selected confocal slices are below the adhesion belts and mostly show lamellar dynamics of normal epithelial cells. At 7’, the transformed cell is above the selected confocal section and presses the AJ of the underlying normal cells (asterisk) down towards the substrate so that it is visible on the confocal slice, at 14’, it breaks through the AJ (arrow) and begins to spread on the substrate, at 21–35’, spreading continues (arrowheads indicate the invasion spot). Scale bar 20 μm.

To visualize migration of transformed cells across epithelial monolayers, we next used IAR-2 cells stably expressing GFP-E-cadherin and IAR-6-1 cells stably expressing mKate2. In IAR-2 cells, GFP-E-cadherin colocalized with the endogenous E-cadherin in stable tangential adherens junctions (S2 Fig). We found that in all cases IAR-6-1 neoplastic cells invaded the IAR-2 monolayer at the boundaries between normal cells (Fig 5A). We observed disruption of E-cadherin-based AJs in IAR-2 cells in the places where the transformed cells penetrated the monolayer (Fig 5B and S7 Video).

### E-cadherin-based AJs between normal and transformed cells affect the migratory behavior of transformed cells

**Fig 6:**
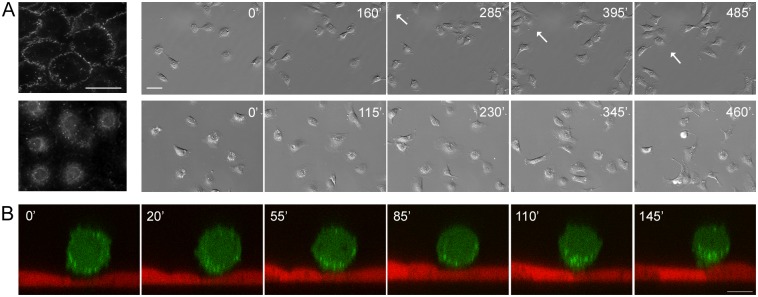
Motile behavior of transformed cells depends on E-cadherin-based AJs. (A) IAR-6-1 (top) and IAR-6-1DNE-E10 (bottom) cells cultured on two-dimensional substrate. Left—immunofluorescent staining for E-cadherin. Scale bar 40 μm. Right—selected frames out of S8 and S9 Videos. IAR-6-1 cells can establish transient cell-cell contacts and are capable of collective migration (group marked with an arrow) (S8 Video) while IAR-6-1DNE cells only touch each other but continue migrating individually. (S9 Video). Scale bar 50 μm. (B) An IAR-6-1DNE-E10 cell on the monolayer of IAR-2 cells. Frames from S10 Video, middle slices out of time lapse confocal Y-stacks. The transformed cell stays rounded and never invades the underlying monolayer over the entire period of observation (compare to Fig 4B). Scale bar 10 μm.

To determine the importance of cell-cell interactions between transformed and normal epithelial cells mediated by E-cadherin, for transformed cell migration, we established sublines of IAR-6-1 cells stably expressing a dominant-negative mutant form of E-cadherin with a W156A mutation in the Ec1 domain that prevented AJ formation [26] (IAR-6-1DNE-E10 and IAR-6-1DNE-H9 clones). Observations of the migratory behavior of IAR-6-1DNE cells on a 2D substrate demonstrated that expression of this E-cadherin mutant dramatically inhibited cell-cell adhesion and collective cell migration (Fig 6A, S8 and S9 Videos). We also found a significant reduction of adhesion of IAR-6-1DNE cells to the IAR-2 epithelial monolayer. The majority of IAR-6-1DNE cells expressing the W156A E-cadherin mutant remained round and did not attach to the surface of the IAR-2 monolayer (Fig 6B and S10 Video).

**Fig 7:**
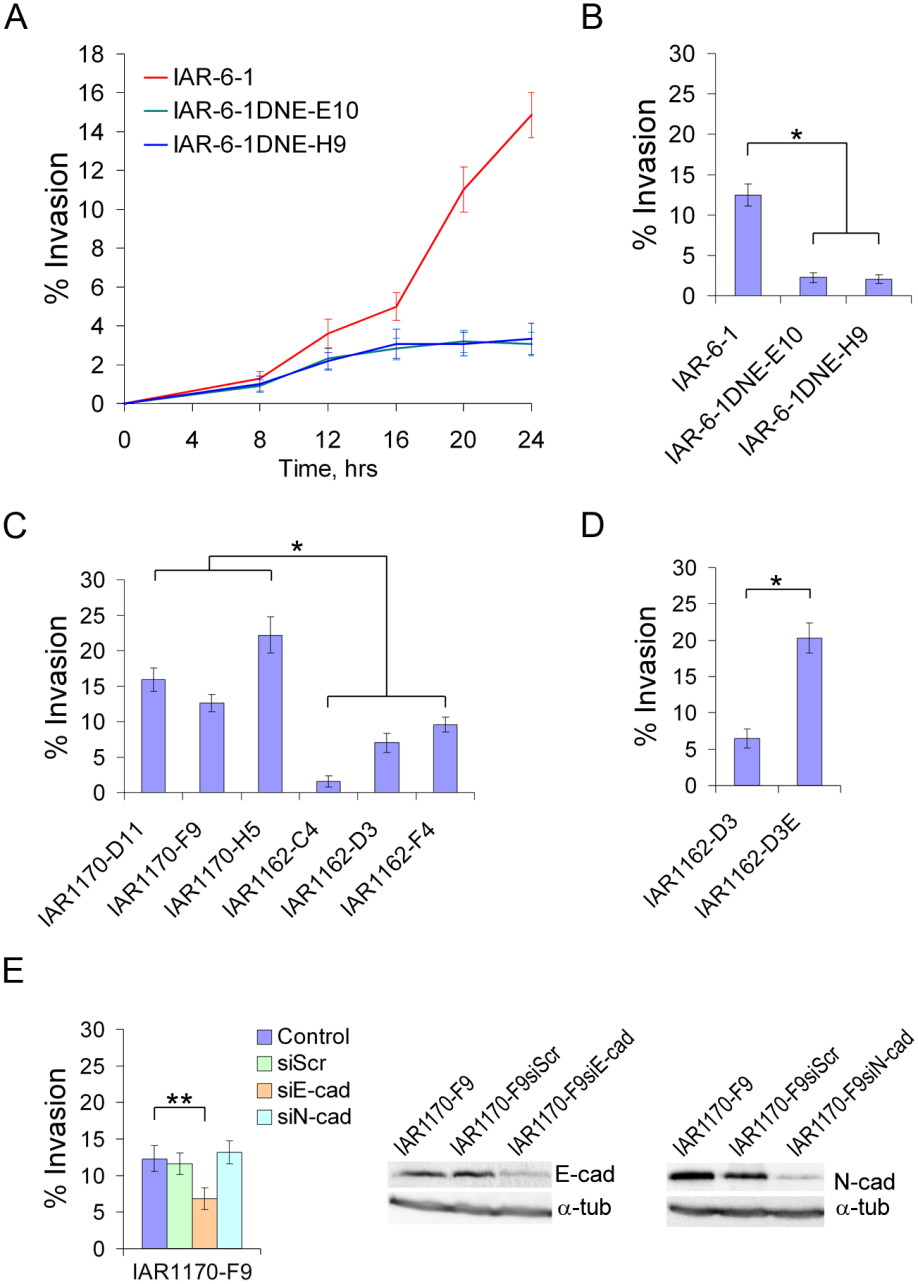
Invasive behavior of transformed cell lines. (A) Dynamics of transepithelial migration of IAR-6-1 and IAR-6-1DNE cells. The diagram shows the percentage of transformed cells that invaded the IAR-2 monolayer and spread on the glass substrate below the monolayer to the number of seeded cells at various time points (mean ± SEM, n = 40). Transfection of a dominant-negative mutant of E-cadherin dramatically decreased the invasion of the epithelial monolayer by transformed cells. (B-D) A comparative study of the invasive behavior of a panel of transformed IAR cells in transepithelial migration assay. The diagrams show the percentage of transformed cells that invaded the IAR-2 monolayer and spread on the glass substrate to the number of seeded cells by 20 hours after seeding (mean ± SEM, n = 30). Asterisks indicate statistically significant differences (Kruskal-Wallis test, *—p‹0.001; **—p‹0.05). (B) IAR-6-1 and IAR-6-1DNE cells stably expressing a dominant-negative mutant of E-cadherin that abolished adhesive cadherin-based interactions. (C) Ras-transformed IAR1170 and IAR1162 clones. Cells that could form E-cadherin-based AJs (IAR1170-D11, IAR1170-F9, IAR1170-H5, IAR1162-D3E) were significantly more invasive than cells that could not (IAR1162-C4, IAR1162-D3, IAR1162-F4). (D) IAR1162-D3 cells and IAR1162-D3E cells stably expressing exogenous E-cadherin. (E) Effect of depletion of E-cadherin or N-cadherin by siRNA on transepithelial migration of IAR1170-F9 cells expressing both E- and N-cadherin.

During 24 h of observation, we compared the dynamics of transepithelial migration of IAR-6-1 line to that of IAR-6-1DNE-E10 and IAR-6-1DNE-H9 clones. The percentage of transformed cells that had invaded the IAR-2 monolayer and spread on the glass substrate below the monolayer to the number of seeded cells at various time points was determined (Fig 7A and 7B). We found that in the absence of cadherin-mediated adhesive interactions, IAR-6-1DNE cells practically lost the ability to invade epithelial monolayer.

In this established cell culture system we performed a comparative analysis of the invasive behavior of a panel of transformed IAR cells. The percentage of cells that had migrated across an IAR-2 monolayer by 20 hours after seeding was determined. Ras-transformed clones: IAR1170-D11, IAR1170-F9, IAR1170-H5 that expressed both E-cadherin and N-cadherin, IAR1162-D3, IAR1162-F4 that lost E-cadherin expression but expressed N-cadherin, and IAR1162-C4 that did not express either E- or N-cadherin were investigated (Fig 7C). We observed statistically significant differences in the percentage of the cells that had invaded the epithelial monolayer between transformed cell lines that formed E-cadherin-based AJs with the normal cells, and the transformed cell lines that did not. Between individual cell lines that formed AJs, as well as between individual cell lines that did not, the differences were more minor. The invasion of the epithelial monolayer by E-cadherin-negative cells of IAR1162-D3 and IAR1162-F4 clones was more pronounced than that of IAR1162-C4 clone, possibly because they express N-cadherin and can form weak heterophilic AJs with IAR-2 cells.

We also compared the invasive behavior of IAR1162-D3 cells and IAR1162-D3E cells that were stably transfected with exogenous E-cadherin (Fig 7D). We found that transfection of exogenous E-cadherin in IAR1162-D3 line resulted in an increase of invasiveness of the epithelial monolayer by these cells that also suggests the important role of E-cadherin-based adhesive interactions between transformed and normal epithelial cells in migration of transformed cells.

In this transepithelial migration assay we also analyzed invasive behavior of IAR1170-F9 clone that had been selectively depleted of E-cadherin or N-cadherin using RNAi technique (Fig 7E). We chose IAR1170 cells as they expressed both E-and N-cadherin. We did not find statistically significant differences in the percentage of the cells that had invaded the epithelial monolayer between control IAR1170 cells and IAR1170 cells transfected with N-cadherin siRNA. With IAR1170-F9 clone transfected with E-cadherin siRNA, we found a partial reduction in the number of cells that had invaded the epithelial monolayer. Thus, our data demonstrate that E-cadherin-based adhesion between neoplastic and normal epithelial cells can regulate motility and invasiveness of the neoplastic cells.

## Discussion

Over the last decade, the main types of cell migration that have a pivotal role in tumor cell dissemination, including individual and collective migration, were investigated [30]. Depending on microenvironment and on the balance between protrusiveness and actomyosin contractility, tumor cells can employ mesenchymal or amoeboid modes of movement, both of which are important for invasion and migration in connective tissue. Arp2/3-mediated polymerization of actin network at the leading edge followed by formation of focal adhesions and pericellular proteolysis of extracellular matrix are key to mesenchymal movement. In amoeboid movement, actomyosin contractility that drives the formation of blebs, allows tumor cells to remodel extracellular matrix in the absence of pericellular proteolysis [30–32]. However, during dissemination in tissues and organs that are preferentially composed of cells, interactions of tumor cells with tissue cells rather than adhesive interactions with the extracellular matrix may be essential. The role of adhesive interactions between neoplastic cells and adjoining normal cells in tumor cell migration has not yet been investigated.

Only a few examples of the involvement of cadherin-based interactions with surrounding cells in cell migration are known. It has been established that migration of zebrafish primordial germ cells within the embryo requires E-cadherin-mediated cell-cell adhesion between germ cells and somatic cells. Dominant-negative mutant of E-cadherin lacking the extracellular domains inhibited the motility of primordial germ cells [33]. An overall down-regulation of E-cadherin as well as its relocation from the cell periphery to the tail of germ cells has been observed at the onset of Drosophila melanogaster germ cell migration, though in this case, the role of cadherin-based interactions with neighboring cells has not been demonstrated [34].

We have previously shown that neoplastic transformation of IAR-2 epithelial cells, induced with mutated Ras or chemical carcinogenes, in case of retention of E-cadherin expression, is accompanied by reorganization of stable, linear E-cadherin-based AJs into dynamic, discontinuous, radial AJs and the disappearance of the marginal actin bundle [23]. Dynamic E-cadherin-based AJs allowed transformed IAR-6-1 cells and IAR1170 cells to detach easily from neighboring cells and migrate individually. At the same time, E-cadherin-based AJs were important for collective migration of IAR-transformed epithelial cells over 2D substrate and also in migration chambers [24].

In this study, we have shown that transformed cells may establish E-cadherin-based AJs with normal epithelial cells and migrate over the confluent epithelial monolayer. When moving over the monolayer, transformed IAR cells accumulated E-cadherin in adhesions that formed in protrusions, and also at the cell sides and the rear. AJs in the extensions were very dynamic. We hypothesize that migration of a neoplastic cell over the epithelial monolayer consists of several sequential steps: extension of protrusions, their adhesion to normal cells using E-cadherin-based adhesions, contraction of the cell body by contractile actomyosin, and translocation of the cell body. The dynamic AJs in protrusions serve to anchor the protrusions to the underlying normal cells, while traction forces exerted by the contractile actomyosin bundles associated with more stable lateral AJs can be instrumental in cell body translocation. We also found that neoplastic cells attached to the epithelial monolayer could not only migrate over the monolayer but also invade it.

To examine whether E-cadherin is involved in the interactions between transformed and normal epithelial cells, we established IAR-6-1 clones expressing a dominant-negative mutant form of E-cadherin with the mutation in the first extracellular domain that prevents homophilic adhesive cell-cell interactions [26] (IAR-6-1DNE). We observed that in the absence of cadherin-based adhesion, IAR-6-1DNE cells practically lost the ability to adhere to IAR-2 cells and invade the IAR-2 epithelial monolayer. This suggests that E-cadherin has an important role in mediation of interactions between neoplastic and normal epithelial cells. In IAR1170 cells expressing both E- and N-cadherin, depletion of N-cadherin with a specific siRNA did not affect transepithelial migration. Transfection with E-cadherin-specific siRNA resulted in a partial reduction of the percentage of transformed IAR1170-F9 cells that had invaded the IAR-2 monolayer.

Our findings show that neoplastic cells invade the epithelial monolayer by disrupting cell-cell contacts between normal epithelial cells. The mechanisms of destruction of the E-cadherin complex in normal epithelial cells during migration of neoplastic cells across the monolayer remain unclear. The studies of interactions between neoplastic and tissue cells have been mostly limited by two models: intra- and extravasation of transformed cells in the transendothelial migration assay [35, 36] and invasion of ovarian cancer cell spheroids into mesothelial monolayer (rev. in [37; 38]), however, both these processes differ significantly from interactions of neoplastic epithelial cells with normal epithelial cells that are the focus of the present study. In a 3D vascular network model of intravasation of tumor cells transcellular invasion with penetration through endothelial cells and paracellullar invasion with disruption of cell-cell contacts between endothelial cells have been observed [39], while we observed only paracellular migration of neoplastic epithelial cells across the epithelial monolayer. The exact mechanisms of intra- and extravasation of tumor cells are not fully understood. It has been established that tumor cells can secrete vasoactive factors such as VEGF contributing to VE-cadherin phosphorylation that destabilizes endothelial cell-cell junctions [40–42]. It has also been shown that metalloproteinase inhibitors significantly reduced tumor cell extravasation [43]. Using broad-spectrum inhibitors of metalloproteinases, marimastat and GM6001, we investigated the potential contribution of metalloproteinases to invasion of monolayer by neoplastic cells but no significant effects were observed (data not shown). It should be noted, however, that in endothelial cells that have to respond quickly to changes in the perivascular microenvironment and intracellular signaling by modulating vascular permeability, the important property of VE-cadherin-based AJs is their plasticity [41, 42, 44]. In contrast, the present study is focused on the stable E-cadherin-based AJs in normal epithelial cells that are essential for the maintenance of epithelial integrity.

In the other studied system modeling peritoneal dissemination of ovarian cancer cells it has been shown that interaction of cancer spheroids with a mesothelial monolayer induces traction forces and promotes mesothelial cell displacement away from the invading spheroid [38]. The precise mechanisms involved in tumor-mesothelial cell interactions remain unclear. The role of cadherin-based adhesion of spheroids and of dynamics of N-cadherin-based AJs in mesothelial cells during invasion of cancer cells into submesothelial matrix was undefined.

Thus, this study gives a new insight into how cadherin-based adhesive interactions with normal cells may be employed by cancer cells to promote their migration. Although EMT with loss of E-cadherin has often been considered as a key program of invasion and metastasis, our observations suggest that non-EMT morphologic transformation in epithelial cells that involves reorganization of E-cadherin-based AJs represents an alternative mode of cancer cell dissemination and may contribute to the plasticity of cancer cell invasion. Our findings suggest that E-cadherin may play a novel important role in dissemination of cancer cells along with its known function in collective invasion. Further studies are required that will be directed towards understanding of molecular mechanisms of motility and invasiveness of cancer cells that involve cadherin-mediated cell-cell adhesion.

## Supporting Information

S1 Fig Tumorigenicity of IAR lines. (TIF)

S2 Fig Colocalization of endogenous and exogenous E-cadherin in AJs. (TIF)

S1 Table Characteristics of normal IAR-2 epithelial cells. (DOCX)

S1 Video In sparse culture, IAR-2 cells form islands and maintain stable cell-cell contacts. (AVI)

S2 Video Transformed IAR-6-1 cells migrate over an IAR-2 epithelial monolayer. Migrating EGFP-expressing IAR-6-1 cells have an elongated fibroblast-like phenotype.
(AVI)

S3 Video A transformed IAR-6-1 cell migrates over an IAR-2 monolayer. The EGFP-expressing IAR-6-1 cell is round, with several dynamic lamellar extensions. Top slices out of confocal Z-stacks.
(AVI)

S4 Video A transformed IAR-6-1 cell forms E-cadherin-based adhesions with underlying normal IAR-2 cells. A GFP-E-cadherin-expressing IAR-6-1 cell on the monolayer of mKate2-expressing IAR-2 cells. The green channel is a “Z-projection” of all three slices in a confocal Z-stack, the red channel is the top slice. At the leading edge of the cell small dot-like E-cadherin adhesions form and disappear, while at the side and the rear of the cell, larger AJs can be seen.
(AVI)

S5 Video A transformed IAR-6-1 cell invades the monolayer of normal IAR-2 cells. An EGFP-expressing IAR-6-1 cell on the monolayer of mKate2-expressing IAR-2 cells, bottom slices out of time lapse confocal Z-stacks (substrate level). A pseudopod invades the monolayer first and shortly afterwards, the cell migrates across the monolayer, spreads, acquires an elongated shape, and migrates underneath the monolayer.
(AVI)

S6 Video A transformed IAR-6-1 cell invades the monolayer of normal IAR-2 cells (confocal XZY view). An EGFP-expressing IAR-6-1 cell on the monolayer of mKate2-expressing IAR-2 cells, middle slices out of time lapse confocal Y-stacks. At 65’, a pseudopod invades the monolayer, following which the cell body migrates across the monolayer.
(AVI)

S7 Video A transformed IAR-6-1 cell invades the epithelial monolayer by disrupting AJs between normal IAR-2 cells. An mKate2-expressing IAR-6-1 cell on the monolayer of GFP-E-cadherin-expressing IAR-2 cells (bottom slices out of confocal Z-stacks, substrate level). At 133’, the IAR-6-1 cell breaks through the AJ and begins to spread on the substrate. The “0’” time point in Fig 5 corresponds to the “119’” time point in the video.
(AVI)

S8 Video IAR-6-1 cell migration over 2D substrate. IAR-6-1 cells can establish transient cell-cell contacts and migrate collectively.
(AVI)

S9 Video IAR-6-1DNE cell migration over 2D substrate. IAR-6-1DNE cells do not form cell-cell contacts and migrate individually.
(AVI)

S10 Video An IAR-6-1DNE cell does not invade the monolayer of normal IAR-2 cells (confocal XZY view). A GFP-expressing IAR-6-1DNE cell on the monolayer of mKate2-expressing IAR-2 cells (middle slices out of time lapse confocal Y-stacks). The transformed cell stays rounded and never invades the underlying monolayer.
(AVI)
