# Supplementary figures and images for: A Novel Role of E-Cadherin-Based Adherens Junctions in Neoplastic Cell Dissemination
Source: PLoS One. 2015 Jul 24;10(7):e0133578. doi: 10.1371/journal.pone.0133578 (PMC4514802; doi:10.1371/journal.pone.0133578)

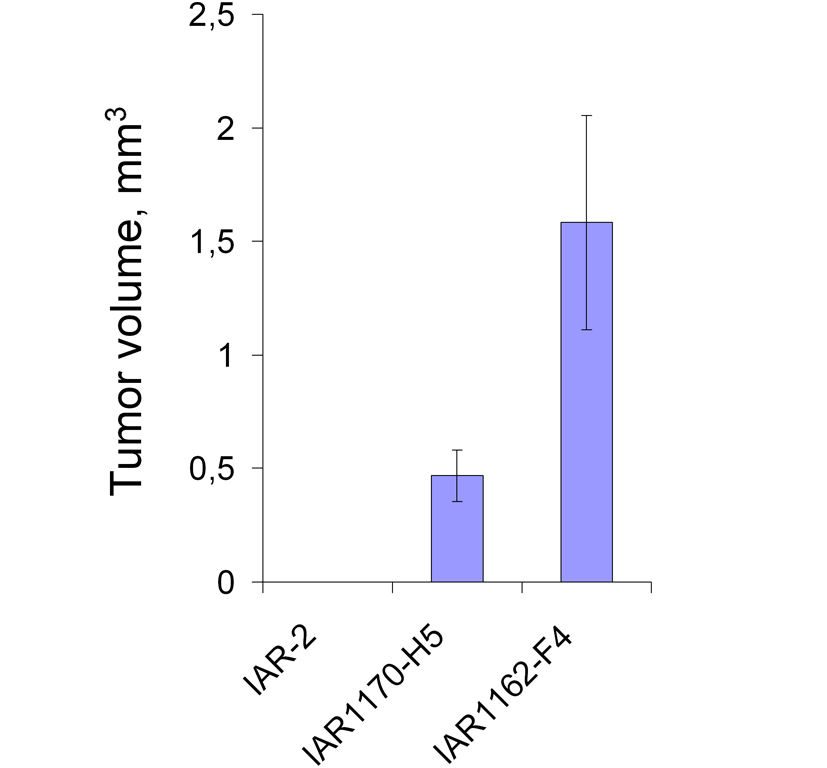

Supplement: S1 Fig — (TIF) [file pone.0133578.s001.tif]

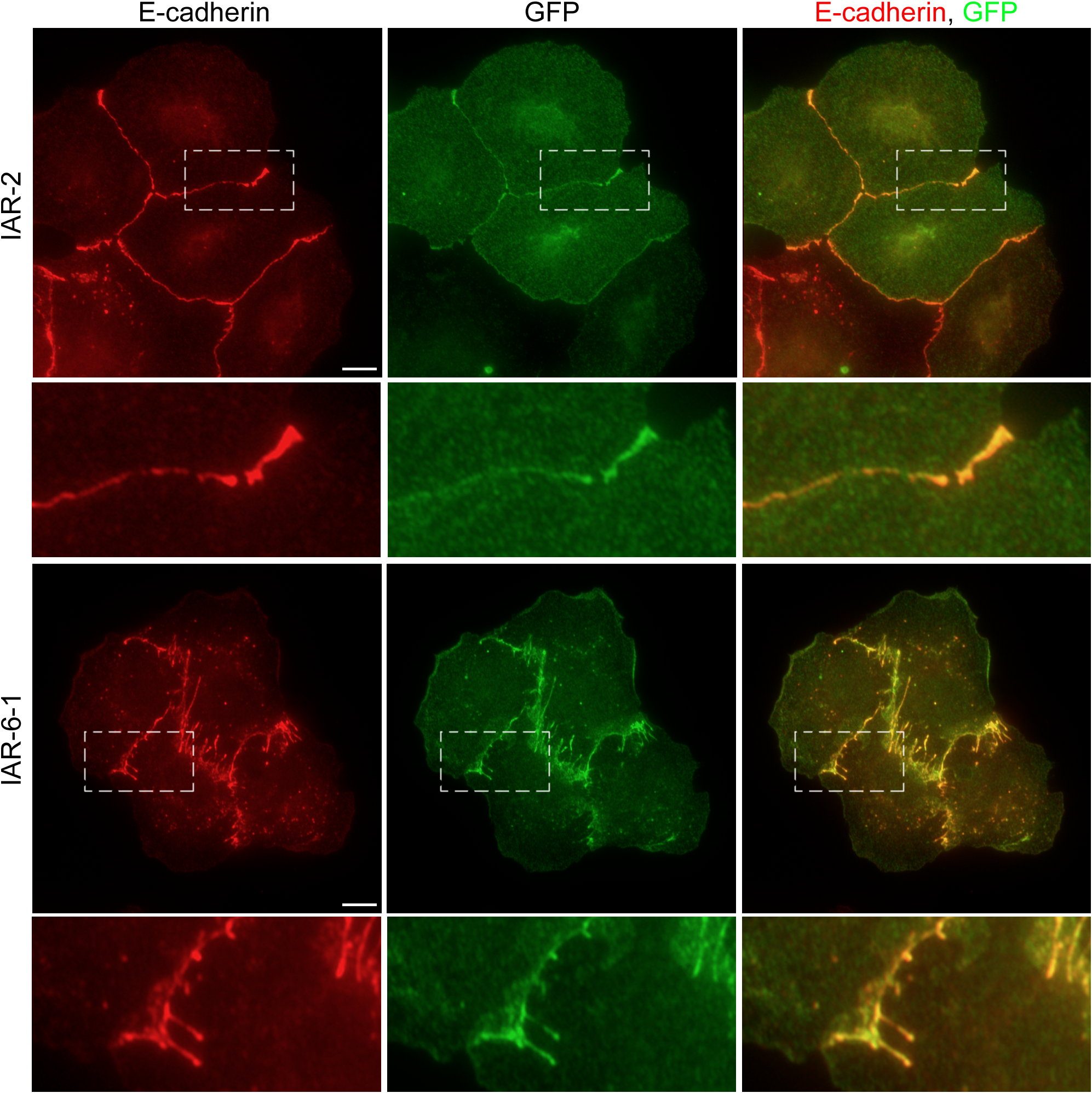

Supplement: S2 Fig — (TIF) [file pone.0133578.s002.tif]
