# Supplementary material for: A Novel Role of E-Cadherin-Based Adherens Junctions in Neoplastic Cell Dissemination
Source: PLoS One. 2015 Jul 24;10(7):e0133578. doi: 10.1371/journal.pone.0133578 (PMC4514802; doi:10.1371/journal.pone.0133578)
Supplement: S1 Table — (DOCX) [file pone.0133578.s003.docx]

**Cell parameters characterizing IAR-2 cells as normal epithelial cells**

| E-cadherin | + |
| --- | --- |
| N-cadherin | - |
| Single cells | Discoid-shaped |
| Sparse culture | Non motile islands |
| Dense culture | Confluent monolayer |
| Adherens junctions | Stable linear adhesions (continuous belts along the cell-cell boundaries) |
| Actin cytoskeleton | Cells have marginal actin bundles |
| Tumorigenicity | Non tumorigenic in nude mice or in or syngeneic rats |
